# A Trihybrid Approach for Enhancing Crude Oil Recovery Using Effervescent‐Tablet‐Based Nanofluids

**DOI:** 10.1002/gch2.202400202

**Published:** 2024-12-19

**Authors:** Naser Ali, Husain Bahzad, Nawaf F. Aljuwayhel, Shikha A. Ebrahim, Abbas T. Hamoud, Hussain Al‐Mazidi, Huda B. Al‐Naser, Mohammad A. Al‐Attar, Sohaib Kholosy, Ayas Al‐Zanki, Mohammad Banyan, Mumayaz Alenezi

**Affiliations:** ^1^ Nanotechnology Applications Program Energy and Building Research Center Kuwait Institute for Scientific Research Safat 13109 Kuwait; ^2^ Department of Chemical Engineering Technology Public Authority for Applied Education and Training Safat 13192 Kuwait; ^3^ Mechanical Engineering Department College of Engineering and Petroleum Kuwait University Safat 13060 Kuwait; ^4^ Improved Oil Recovery Program Petroleum Research Center Kuwait Institute for Scientific Research Safat 13109 Kuwait

**Keywords:** enhanced oil recovery, MWCNT, oil composition, thermal recovery, thermophysical property

## Abstract

While nanofluids can contribute enormously to enhanced oil recovery (EOR) in the upstream sector, conventional nanofluids are produced using complex equipment and expertise, which is somewhat limiting. To address this issue, herein, the use of effervescent‐tablet‐based nanofluids for EOR is reported. Tablets are formed by mixing and consolidating multi‐walled carbon nanotubes, surfactants, and effervescent agents. Both tablet‐based and conventional nanofluids are produced and then characterized for their thermal conductivities and dispersion stabilities. Thirteen recovery scenarios are investigated using a core flooding system, and include the use of single conventional fluids, steam and hot water cycles, steam with effervescent‐tablet‐based nanofluids, and steam with conventional nanofluids. Conventional or effervescent‐tablet‐based nanofluids are revealed to double the amount of extracted oil compared with other methods used in the recovery process. Tablet‐ and conventional‐nanofluid‐based recovery cycles provide 42.70% and 42.56% recovered oil, respectively, whereas conventional fluids and their cycles only extract 16.10% and 17.76%, respectively. The concentration and stability of the dispersed nanomaterial significantly affect the amount, properties, and composition of the recovered oil. Employing nanofluids composed of highly concentrated effervescent agents results in more short‐chain hydrocarbons, which indicates that effervescent‐tablet‐based nanofluids are promising for EOR use, particularly because no infrastructure modifications are required.

## Graphical abstract

Nanofluids improve oil recovery: Conventional nanofluids are intensively explored for extracting crude oil. This study finds that using effervescent‐tablet‐based nanofluids is more effective and convenient for this task. They overcome their counterpart's performance as well as other thermal flooding methods and do not require specialized equipment to fabricate the suspension, making them a promising alternative to conventional nanofluids.

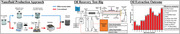

## 1 Introduction

Population growth has led to global increases in energy demand. For instance, the energy demands of Belgium, Russia, and France have risen by 11.3%, 9.4%, and 7.7%, respectively, between 2020 and 2021.^[^ ^1^ ^]^ Countries are continual looking for new resources and developing existing ones to fulfill their energy requirements, which includes exploring depleting nonrenewable sources, such as new crude oil and natural gas reservoirs, and expanding the use of renewable sources such as solar and wind energies. However, the feasibility of any energy source depends on a number of factors, including net cost, availability, and environmental impact.^[^ ^2^, ^3^, ^4^, ^5^, ^6^, ^7^ ^]^ Despite the wide variety of available energy resources, completely disengaging our reliance on crude oil and its byproducts remains challenging, which is mainly ascribable to many economies having structured and developed their industries to uses such energy sources, whose high energy densities and portability are ideal for combustion engines (e.g., automotive). Moreover, large investments in existing infrastructure (e.g., refineries, storage facilities, and pipelines) in terms of capital and time, and challenges associated with alternative energy sources are yet to be fully resolved.^[^ ^8^, ^9^, ^10^, ^11^ ^]^ Therefore, further developing and implementing innovative solutions that help to improve oil recovery from pre‐existing wells is important, especially because current methods can only recover between ≈40% and ≈60% of the oil, depending on type (e.g., light, medium, and heavy crude oil), underground geological formation (e.g., sand, carbonate, shale, etc.), and recovery method used (e.g., thermal and/or chemical).^[^ ^12^ ^]^ Notably, chemical recovery approaches involve adding chemicals to the injected fluid to reduce interfacial tension and/or improve hydrocarbon‐production sweep efficiency.^[^ ^13^, ^14^, ^15^, ^16^ ^]^ Crude oils exist in different forms or grades depending on their geographical origin and chemical composition. According to the measurement standards provided by the American Petroleum Institute (API), crude oil can be classified into five main categories:^[^ ^14^, ^17^, ^18^, ^19^, ^20^ ^]^

1) light crude oil, API gravity > 31.1°;
2) medium crude oil, API gravity between 31.1° and 22.3°;
3) heavy crude oil, API gravity between 22.3° and 20°;
4) extra heavy crude oil, API gravity between 20° and 10°; and
5) bitumen, API gravity < 10°

It is worth noting that API gravity measurements are usually conducted at 15.6 °C and that light crude oil has the lowest density among the various categories; consequently, it is among the most valuable classes of crude oil because it yields higher percentages of high‐value light products when refined.

Most initially reported studies examined the use of hot‐water flooding and steam injection for enhanced oil recovery (EOR), after which chemicals were introduced into the procedure to further improve the process. Normally, the type of fluid used for EOR is determined based on the type of target crude oil, where water, water‐alternating gas (WAG),^[^ ^21^ ^]^ miscible gas,^[^ ^22^ ^]^ or polymers^[^ ^23^ ^]^ are preferably used for light or low‐viscosity oils, and steam, hot water, or gas are generally considered to be more‐suitable working fluids for heavy or highly viscous crude oils.^[^ ^24^, ^25^, ^26^ ^]^ Shu and Hartman^[^ ^27^ ^]^ used a thermal compositional simulator to investigate the contribution of steam flooding to visbreaking, a process that permanently reduces viscosity and is typically used when refining crude oils. The authors concluded that visbreaking improves oil recovery by creating a transition zone; consequently, an ideal visbreaking rate can be achieved by tuning the steam temperature, quality, and injection approach. Ziegler^[^ ^28^ ^]^ developed analytical models for assessing the injection of steam/hot water into fractured reservoirs, with both diatomite and shale reservoir rock samples used in the analysis, and capillary imbibition and thermal expansion considered as mechanisms of interest during model development. The authors concluded that steam injection yielded better results than hot‐water injection, with particular emphasis on diatomite reservoirs that contain lighter crude oil. Rao et al.^[^ ^29^ ^]^ studied how miscible gas flooding affects wettability; their methodology included core flooding systems with different wettabilities, namely water‐wet, oil‐wet, and mixed wettability systems. Cyclic CO_2_ miscible flooding was implemented, in which CO_2_ slugs of a specific size were intermittently injected during the water flooding process, which revealed that overall CO_2_ miscible flooding positively altered wettability, as water‐wet systems remained relatively unchanged, whereas mixed‐wettability and oil‐wet systems exhibited higher oil recovery rates. Srivastava et al.^[^ ^30^ ^]^ experimentally studied a mature Weyburn reservoir located in southeastern Saskatchewan, Canada using CO_2_ miscible flooding as the EOR methodology. Several Weyburn oilfield samples were collected for use in laboratory experiments and recording purposes, with minimum miscibility pressure (MMP) and pressure–volume–temperature (PVT) properties used as analysis parameters. Core‐flooding experiments revealed that CO_2_ miscible flooding led to lower Weyburn‐oil viscosity and oil swelling, which led the authors to conclude that CO_2_ miscible flooding is a promising EOR methodology for the Weyburn reservoir. Al‐Otaibi et al.^[^ ^31^ ^]^ investigated the use of miscible flooding for recovering oil from carbonate reservoirs using different carbonate rock cores obtained from the reservoir of interest and three supercritical‐CO_2_‐injection modes: continuous CO_2_ flooding, WAG injection, and tapered WAG injection. These protocols were used to design five core‐flooding experiments for assessing the viability of each injection mode. The authors concluded that considerable performance differences exist among the injection modes. Although better conformance control and recovery factors were obtained during WAG compared to continuous CO_2_ injection, vertical CO_2_ injection delivered the best performance.

Unfortunately, the conventional processes described above have reached a point beyond which no significant improvements can be achieved; hence, new technological routes must be adopted to overcome these limitations. Nanotechnology, as an emerging modern technology, offers a promising approach in conjunction with current EOR methods. One way of implementing nanotechnology in the EOR process involves replacing conventional currently used working fluids with nanofluids, which were initially introduced by Masuda et al.^[^ ^32^ ^]^ in 1993 and subsequently defined by Choi and Eastman^[^ ^33^ ^]^ in 1995. Nanofluids are fabricated by dispersing nanoscale particles in a host base fluid incapable of dissolving them. Consequently, the dispersed nanomaterial alters the properties of the liquid and the surrounding geometrical structures when a base fluid (usually water) containing these dispersed particles is used in the flooding process. As such, they can alter the wettabilities of the surrounding rocks, reduce the viscosity of the oil, and increase the underground build‐up pressure in a manner that depends on the type of material used and nature of the underground formation. Moreover, various studies have reported enhanced oil recoveries that are up to 30% higher than those obtained using conventional methods by simply using a nanofluid as the working fluid in the EOR process.^[^ ^34^, ^35^ ^]^ For example, Resasco et al.^[^ ^36^ ^]^ reported that a carbon‐based nanofluid containing an anionic surfactant can reduce interfacial tension by 70% compared that obtained using pure water with the same amount surfactant, which highlights the primary role played by the dispersed nanomaterial in the EOR process. Soleimani et al.^[^ ^35^ ^]^ investigated how dispersions of carbon nanotubes affect the oil‐recovery process. Two pore volumes were considered for brine and the nanofluid (as the working fluids) in the core‐flooding setup, which revealed that a 0.3‐wt.% carbon‐based suspension is capable of recovering 18.57% more oil than its conventional counterpart. Li et al.^[^ ^37^ ^]^ investigated a newly developed carbon‐based nanofluid for EOR. Nanofluids containing 0.001–0.1 wt.% of the carbon‐based nanomaterial were produced, with the 0.1 wt.% nanofluid and brine revealed to recover 38.3% and 16.8% of the oil, respectively. These results demonstrate that the developed nanofluid is capable of recovering more than twice the amount of oil that the conventional fluid. Ghalamizade et al.^[^ ^38^ ^]^ conducted a field study into the use of carbon‐based nanomaterials for improving oil recovery and found that the carbon‐based suspensions were superior to conventional liquids in the EOR process. Specifically, nanofluid flooding led to a 20% higher recovery than that obtained using water flooding.

Two approaches are generally used to produce nanofluid suspensions. The first route is referred to as the single‐step (or one‐step) approach, with the second referred to as the two‐step approach. The former approach, in which nanoparticles are formed and dispersed within the host liquid in a single stage, is advantageous for the following reasons: 1) the suspension exhibits high physical dispersion stability and 2) it avoids the necessity of managing dry powders, including their transportation and storage. However, this production approach is always associated with residuals that are challenging to eliminate owing to incomplete reactions; hence, it can only be used to synthesize certain combinations of nanoparticles and liquids. In addition, the equipment used in the one‐step‐production approach is typically complicated and costly. On the other hand, the two‐step approach uses pre‐prepared powders, which are then added to a non‐dissolving base fluid and dispersed using a mixing device, such as a magnetic stirrer, homogenizer, or ultrasonicator. This approach has advantages that include the ability to produce any type of nanofluid, ease of management by users with average skills, the wide global availability of commercial powders, and its ability to be used in small‐ and large‐scale production. Consequently, this production approach is favored by many scholars working in the nanofluid field. Nevertheless, the mixing device is a key factor in the two‐step suspension‐production process; hence, this fabrication method is challenging to use in remote areas where electrical sources are limited. Furthermore, the dispersions prepared using this two‐step approach are less physically stable than those prepared using the one‐step method; however, this situation can be improved to a certain extent by including surfactants in the mixture at the fabrication stage or by surface‐functionalizing the particles.

**Figure 1:**
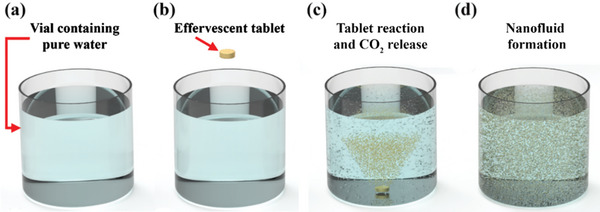
Reaction of an effervescent‐tablet in a liquid. a) Vial with pure water, b) tablet prior to being placed in the water, and c,d) formation of CO_2_ bubbles and accompanying nanomaterial dispersion before and after full tablet dissolution, respectively.

In addition to the two discussed suspension‐production methods, new nanofluid‐production approaches were reported by Ali et al. in 2023^[^ ^39^ ^]^ and Alsayegh et al. in 2024^[^ ^40^ ^]^ for fabricating dispersions directed toward thermal applications, with effervescent‐tablet technology having been integrated with suspension science to fabricate carbon‐based nanofluids for use in thermal applications. Moreover, this approach was demonstrated to produce cost‐effective suspensions with superior thermal properties than those produced conventionally. The effervescent‐tablet‐based nanofluid is prepared by the end‐user, who only needs to add effervescent‐tablets to a base fluid and then wait for ≈12 min for the generated CO_2_ bubbles to completely disperse the particles within the host liquid, thereby completing nanofluid production. Consequently, only minimal experience is required by the end user to prepare such suspensions. The effervescent‐tablet‐based nanofluid‐fabrication process is illustrated in **Figure** **1**, and has been extended to include coatings,^[^ ^39^ ^]^ crystal‐layer‐growth,^[^ ^41^ ^]^ quenching,^[^ ^42^ ^]^ and hydrogen‐production^[^ ^43^, ^44^ ^]^ applications.

Regardless of the approach used to fabricate a nanofluid (i.e., one‐step, two‐step, or effervescent‐tablet), delivering optimum thermophysical properties depends on ensuring a high level of dispersed‐particle stability. Various methods have been used to determine the physical stability of a fabricated nanofluid, including zeta‐potential analysis, dynamic light scattering (DLS), sedimentation photographic capturing, the 3‐ω approach, transmission electron microscopy (TEM), scanning electron microscopy (SEM), centrifugation, spectroscopy, and particle‐size analysis.

Among the abovementioned approaches, sedimentation photographic capturing is considered to be among the most accurate methods for determining the stability of particle dispersion in a suspension under static conditions. However, two (or more) of the abovementioned techniques are preferably employed to ensure that the suspension is physically stable.

**Figure 2:**
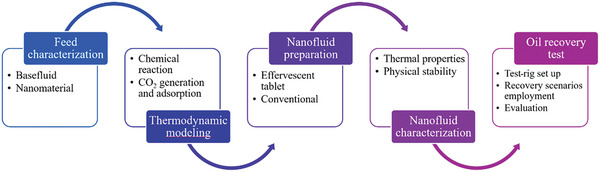
Summarizing the procedure used in this research study.

For the first time, this study investigated the use of effervescent‐tablet‐based nanofluids for improving crude oil recovery. To the best of our knowledge, such a study has never been reported, which highlights its novelty. Viscous and capillary forces form the scientific basis of this type of EOR research; the examined effervescent‐tablet‐based nanofluids affect both forces,^[^ ^45^, ^46^, ^47^, ^48^ ^]^ which justifies their use as flooding fluids. Suspensions were formed using multi‐walled carbon nanotubes (MWCNTs) owing to their outstanding heat‐transfer capabilities compared to other existing nanomaterials.^[^ ^49^ ^]^ Effervescent‐tablets were first prepared by homogeneously mixing MWCNTs, sodium dodecyl sulfate (SDS), NaH_2_PO_4_, and Na_2_CO_3_ at different weights to afford five different powders, which were subsequently consolidated into tablets and stored in an oil‐recovery test rig for later use. Thirteen oil‐recovery scenarios were applied to the test rig, with results compared in terms of recovered‐oil percentage and composition. These scenarios included conventional fluid, steam, and hot‐water cycles, steam and effervescent‐tablet‐based nanofluid cycles, and steam and conventional nanofluid cycles, as outlined in **Figure** **2**. This study provided promising results, which is believed to be particularly useful for enhancing crude‐oil recovery if adopted by the oil industry, mainly because, unlike conventional nanofluids, producing effervescent‐tablet‐based nanofluids eliminates the need for external mixing devices and sophisticated suspension production, thereby significantly simplifying the nanofluid‐fabrication process. These benefits also extend beyond the EOR field, as effervescent‐tablet‐based nanofluids are useful in materials science,^[^ ^50^ ^]^ heat‐transfer applications,^[^ ^51^ ^]^ water‐desalination systems,^[^ ^52^ ^]^ biomedical engineering,^[^ ^53^ ^]^ hydrogen production,^[^ ^54^ ^]^ and coatings applications.^[^ ^55^ ^]^

## 2 Experimental Procedures

### 2.1 Materials and Consumables

MWCNTs (inner diameter (ID): 5–10 nm, outer diameter (OD): 30–50 nm, axial length: 5–15 µm, and purity >95 wt%), were obtained from SkySpring Nanomaterials, while ReagentPlus NaH_2_PO_4_ (≥99.0 wt%), Na_2_CO_3_ (≥99.5 wt%), and SDS (≥98.5 wt%) were obtained from Sigma–Aldrich and used as feedstocks for fabricating the effervescent‐tablets. Furthermore, transparent glass vials (OD: 4.0 cm, ID: 3.68 cm, and height: 9.5 cm) were purchased from Glass Solutions Limited. Moreover, three types of water were used, namely distilled water (DW), treated seawater (TSW), and modified DW. The DW was produced using a ZIQ7000T0C ultra‐pure water‐generation unit (Merck Millipore), while the TSW was provided by a seawater‐treatment plant in Subiya, Kuwait. In contrast, the modified DW was prepared to reflect well‐formation water, which was provided by the Kuwait Oil Company (KOC). The specifications of the modified DW, including its total dissolved solids (TDS), are provided in **Table** **1**.

**Table 1:** Specifications of the modified DW and well‐formation water obtained from the KOC.

Component	Units	KOC water	As‐modified DW	Difference error %
Calcium	mg L^−1^	1675	1647.5	0.83
Magnesium	mg L^−1^	1450	1414	1.26
Sodium	mg L^−1^	10200	10182	0.09
Potassium	mg L^−1^	135	149	4.93
Chloride	mg L^−1^	22550	22917	0.81
pH	–	7.1	6.5	4.41
Density @ 25 °C	g cm^−3^	1.024	1.023	0.03
TDS (Measured)	mg L^−1^	36560	36400	0.22

### 2.2 Powder Analysis

**Figure 3:**
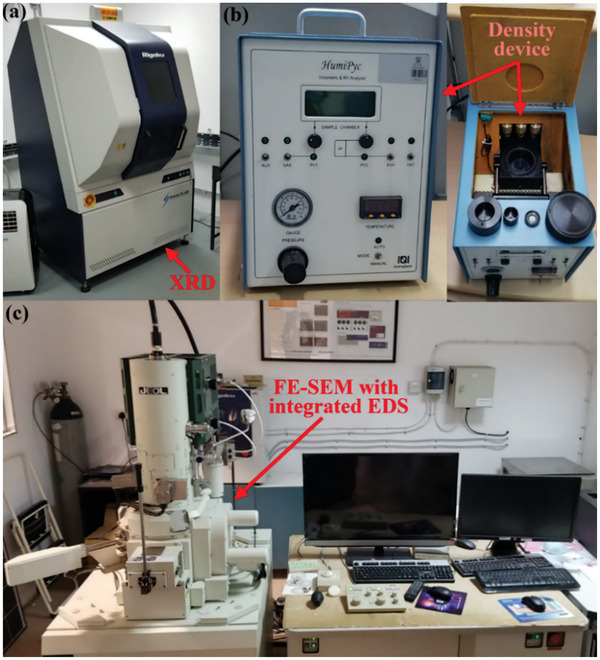
Powder‐analysis instruments. a) X‐ray diffractometer, b) pycnometer, multi‐technique volumetric analyzer, and c) FE‐SEM system with its augmented EDS unit.

The as‐received powders were initially characterized before being used in common literature‐reported methods,^[^ ^56^ ^]^ which involved determining particle morphology, purity, elemental content, and density. The instruments used to analyze the powders are shown in **Figure** **3**. The elemental contents of the as‐supplied powders were determined by X‐ray diffractometry (XRD) using a SmartLab diffractometer (Rigaku) at an operating power of 9 kW and commercial SmartLab Guidance software. A CuK_α_ X‐ray source was used with Bragg peaks acquired in the 20–80° 2θ range at 1° min^−1^ using a 0.1° incidence beam. Furthermore, field‐emission SEM (FE‐SEM; JSM‐IT700HR, JEOL) augmented with energy dispersive X‐ray spectroscopy (EDS) was used to examine particle morphology, apparent particle size, and powder purity aided by commercial InTouchScope (version 1.12) software. It should be noted that the as‐received powder samples were poorly conductive and were consequently coated with gold to enhance FE‐SEM resolution (the MWCNTs were not coated as they are well known to be highly conductive).^[^ ^49^ ^]^ FE‐SEM images of the examined samples were subsequently acquired in secondary electron mode at a working distance of 10 mm and an accelerator voltage of 5 kV, and elemental distributions and corresponding percentages were mapped by switching to the EDS system. Moreover, the density of the as‐supplied MWCNTs (ρ_*MWCNTs*_), which is required for calculating the volume percentage of the dispersed nanomaterial, was determined using a gas pycnometer, multi‐technique volumetric analyzer (Model 1, HumiPyc). Accordingly, 1.5 g of the MWCNT mass (*m_MWCNTs_*), which was measured using an ae‐ADAM PW 214 (Adam Equipment) analytical balance (accuracy: ±0.2 mg, readability: 0.1 mg), was placed in the HumiPyc device. The former device measures the volume of the sample (i.e., MWCNT volume (*V_MWCNTs_*)), after which both measured parameters are used to calculate the ρ_*MWCNTs*_ using Equation (1):

(1) $$\rho_{MWCNTs}=\frac{m_{MWCNTs}\;}{V_{MWCNTs}}$$

which led to a ρ_*MWCNTs*_ value of ≈2.097 g cm^−3^.

### 2.3 Starting Liquid Properties and Characteristics

Seawater (from the Subiya Treatment Plant, Kuwait) and DW were used as the liquids in the EOR experiments, with DW used as the base fluid to prepare the suspensions. The apparent color, pH, properties, mineral content, and total dissolved solids were determined visually, experimentally, and theoretically, the results of which are presented in **Table** **2**. It should be noted that all experiments were conducted at 25 °C employing measuring devices with auto‐calibration features, with a hot‐wire‐type thermal‐conductivity device used according to the ASTM D7896‐14 standard.^[^ ^57^ ^]^ The tabulated thermal conductivity and pH values are based on the averages of three measurements, as commonly reported in the literature,^[^ ^58^, ^59^ ^]^ and TDS calculations are based on the net values of the measured alkalinity and dissolved solids in the examined liquid sample.^[^ ^60^ ^]^

**Table 2:** As‐obtained properties of treated seawater and distilled water.

	Units	Liquid	
		Treated seawater	Distilled water
Appearance	–	Colorless	Colorless
Viscosity	mPa.s	1.19	0.89
Thermal conductivity	W m^−1^ K^−1^	0.609	0.606
Specific heat capacity	kJ kg^−1^ K^−1^	4.009	4.182
Conductivity	mS cm^−1^	67.4	<0.001
Density	g cm^−3^	1.03	0.98
pH	–	7.9	6.8
Carbonate alkalinity	mg L^−1^	0	0
Bicarbonate alkalinity	mg L^−1^	221.15	2.86
Hydroxide alkalinity	mg L^−1^	0	0
Total alkalinity	mg L^−1^	221.15	2.86
Barium	mg L^−1^	0	0.15
Boron	mg L^−1^	3	<0.01
Calcium	mg L^−1^	541	<0.01
Chloride	mg L^−1^	23250	<0.01
Iron	mg L^−1^	0.06	<0.01
Lithium	mg L^−1^	0.28	<0.01
Magnesium	mg L^−1^	1890.70	<0.01
Potassium	mg L^−1^	610	<0.01
Silicon	mg L^−1^	0.42	<0.01
Sodium	mg L^−1^	13750	<0.01
Strontium	mg L^−1^	8.3	<0.01
Sulfate	mg L^−1^	3650	<1
TDS (calculated)	mg L^−1^	43924.91	3.02

### 2.4 Effervescent‐Tablet Production

Effervescent‐tablets were produced by homogeneously mixing MWCNTs, SDS, NaH_2_PO_4_, and Na_2_CO_3_ at 1:1:6.8:2 (Case 1), 1:1:13.6:4 (Case 2), 1:1:20.4:6 (Case 3), 1:1:40.6:11.93 (Case 4), and 1:1:203:59.67 (Case 5) weight ratios to form five different batches. The MWCNT/SDS weight ratio was maintained at 1:1 in all five cases; this ratio was selected based on stability studies reported by Ali et al.,^[^ ^39^, ^61^ ^]^ who revealed the optimum combination for stabilizing carbon‐based suspensions. The mass of each material included in the five powder mixtures is listed in **Table** **3**; the mass of each component was selected to examine how EOR is affected by the dispersed‐particle mixture (by fixing the amount of nanomaterial and varying the quantity of effervescent agent; Cases 1–3) and the MWCNTs (by fixing the amount of effervescent agent and varying the quantity of nanomaterial; Cases 4 and 5).

**Table 3:** Mass of each material in each mixed powder batch used to fabricate effervescent‐tablets.

Case	Material weight				Total powder weight (g)
	MWCNTs (g)	SDS (g)	NaH_2_PO_4_ (g)	Na_2_CO_3_ (g)	
1	2.098	2.098	14.264	4.196	22.655
2	2.098	2.098	28.527	8.392	41.114
3	2.098	2.098	42.802	12.587	59.585
4	1.055	1.055	42.802	12.587	57.498
5	0.211	0.211	42.802	12.587	55.811

**Figure 4:**
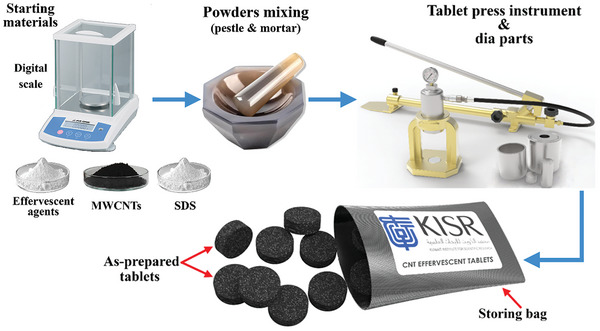
Depicting the process used to produce effervescent‐tablets.

It is important to note that the masses of the powder‐mixture components in Case 1 were selected based on 0.1 vol% MWCNT and a MWCNT/SDS/NaH_2_PO_4_/Na_2_CO_3_ weight ratio of 1:1:6.8:2. The MWCNT and SDS masses were fixed at those used in Case 1, while the NaH_2_PO_4_ and Na_2_CO_3_ masses were both doubled and tripled to afford Cases 2 and 3, respectively. In addition, the masses of NaH_2_PO_4_ and Na_2_CO_3_ used in Case 3 were fixed, while the MWCNT and SDS masses were reduced to afford the 0.05 and 0.01 vol% suspensions used in Cases 4 and 5, respectively. It is worth noting that volume percentages were calculated based on a base fluid of 1 L, and that both NaH_2_PO_4_ and Na_2_CO_3_ are considered to be the effervescent agents that mainly generate CO_2_ gas when reacted in water. Furthermore, the as‐received powders were mixed for 15 min using a pestle and mortar. Portions of the as‐mixed powders were then placed in a pneumatic compression instrument (SSP‐10A, SHIMADZU) with a 25‐mm inner diameter and compressed into tablets at 100 kN, with 2.041, 3.704, 5.368, 5.180, and 5.028 g of respective powder used to form the tablets used in Cases 1–5, respectively. The tablets were discharged from the die and stored in isolated containers for later use. **Figure** **4** shows the process used to fabricate the effervescent‐tablets.

### 2.5 Thermodynamically Modeling Effervescent‐Tablets

**Figure 5:**
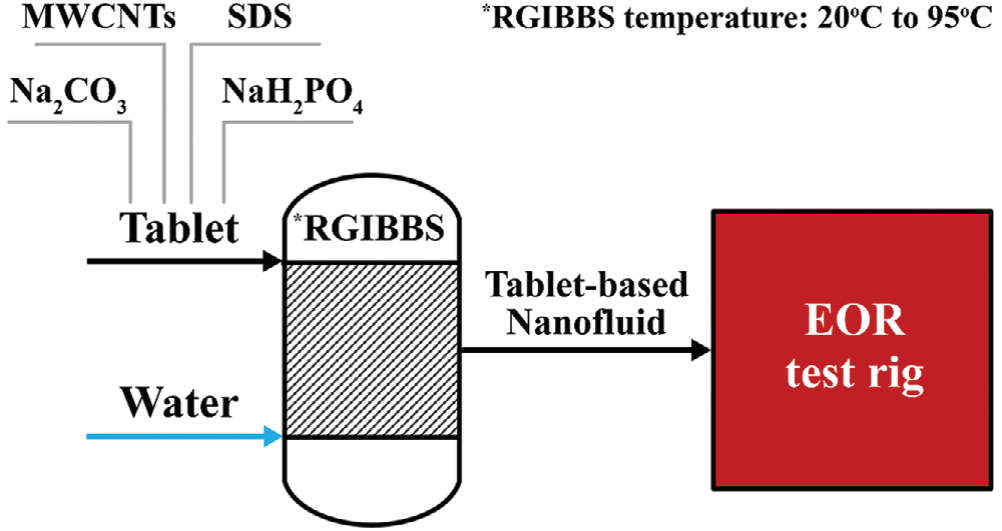
Thermodynamic model for the effervescent‐tablets fabricated for enhanced oil recovery.

A thermodynamic model was developed to determine the components present in the solution formed following dissolution of the effervescent‐tablet in water,^[^ ^39^ ^]^ as illustrated in **Figure** **5**. Two primary chemical reactions generate effervescence, namely:

(2) $$Na_{2}CO_{3}+\mathrm{Na}H_{2}PO_{4}\to \mathrm{NaHC}O_{3}+Na_{2}\mathrm{HP}O_{4}$$

(3) $$\mathrm{NaHC}O_{3}+\mathrm{Na}H_{2}PO_{4}\to Na_{2}\mathrm{HP}O_{4}+CO_{2}+H_{2}O$$

The two effervescent agents are the two reaction components in Equation (2) (i.e., Na_2_CO_3_ and NaH_2_PO_4_). The amount of each component involved in Equations (2) and (3) was calculated assuming that the solution reaches equilibrium. Furthermore, calculations were performed across the 20–95 °C temperature range in increments of 5 °C. The equilibrium composition of the solution was determined based on the calculated amounts, with the process modeled using a Gibbs reactor (RGIBBS) in ASPEN PLUS V.12 software. The Gibbs free energy of the solution was minimized and the equilibrium amounts of the solution components subsequently calculated.

Both the MWCNTs and SDS were considered to be inert in the RGIBBS reactor, as they only affect the process at high temperatures (i.e., > 327 °C),^[^ ^39^ ^]^ which is not the case in the current study. However, MWCNTs can adsorb CO_2_ at a rate of 7 mg_CO2_ g^−1^ at 25 °C.^[^ ^62^ ^]^ Therefore, the amount of CO_2_ adsorbed by the MWCNTs at 25 °C was calculated using Equation (4):

(4) $$m_{CO_{2,a}}=m_{CO_{2,g}}\times C_{MWCNT}$$

where $m_{CO_{2,\;a}}$ is the amount of CO_2_ adsorbed, $m_{CO_{2,\;g}}$ is the amount of CO_2_ generated by the reaction depicted in Equation (3), and *C_MWCNT_* is the full capacity of the MWCNTs to adsorb CO_2_. Furthermore, the model was simulated using different masses of Na_2_CO_3_ and NaH_2_PO_4_, MWCNT, and SDS to explore the influence of various material‐mass combinations on the amount of CO_2_ generated via the reaction shown in Equation (3). The mass concentration of the effervescent agents in the effervescent solution was determined using Equation (5):

(5) $$C_{i}=\frac{m_{i}}{V}\;$$

where *C_i_* is the concentration of the effervescent agent in solution (g L^−1^), *m_i_* is the mass of the effervescent agent in solution (g), and *V* is the solution volume. Table 3 lists the combinations of material masses investigated. Moreover, the effect of reaction temperature on the equilibrium conversion of NaHCO_3_ was modeled according to Equation (3), with Equation (6) used to calculate the equilibrium conversion of NaHCO_3_:

(6) $$X_{NaHCO_{3}}=\frac{n_{NaHCO_{3},I}-n_{NaHCO_{3},O}}{n_{NaHCO_{3},I}}\;$$

where $n_{NaHCO_{3},I}$, and $n_{NaHCO_{3},O}$ are the amount (mol) of NaHCO_3_ generated by the process shown in Equation (2), and the amount (mol) of NaHCO_3_ remaining at the end of the reaction depicted in Equation (3) at equilibrium.

### 2.6 Nanofluid Preparation and Characteristics

#### 2.6.1 Suspension Production

Two types of suspension were prepared: effervescent‐based and conventional. To produce the effervescent‐tablet‐based suspensions, eleven of the as‐fabricated effervescent‐tablets were dropped into the liquid container attached to the test rig, which holds 1 L of DW (at 25 °C), to commence the reactions that form the Case 1–5 suspensions. Once complete, the container hosting the suspension was heated to 98 °C before use as a working fluid in the test rig. On the other hand, conventional suspensions were fabricated by externally adding MWCNTs and SDS (1:1 weight ratio) to DW (four batches, each containing 0.25 L DW to afford a total of 1 L). The mixture was then stirred using a magnetic stirrer for 5 min, after which the content was dispersed in a controlled temperature‐bath sonicator (25 °C) for 30 min to form 0.01, 0.05, and 0.10 vol% nanofluids. The volume percentage of the dispersed MWCNTs was calculated assuming a base fluid volume (*V_bf_*) of 1 L through mixing theory,^[^ ^63^, ^64^ ^]^ using Equation (7) and (8):

(7) $$\mathrm{vol}\%=\frac{V_{MWCNTs}\;}{V_{bf}+V_{MWCNTs}\;}\;\times 100$$

(8) $$\mathrm{vol}\%\;=\frac{\frac{m_{MWCNTs}\;}{\rho_{MWCNTs}}\;}{V_{bf}+\frac{m_{MWCNTs}\;}{\rho_{MWCNTs}}\;}\;\times 100$$

**Figure 6:**
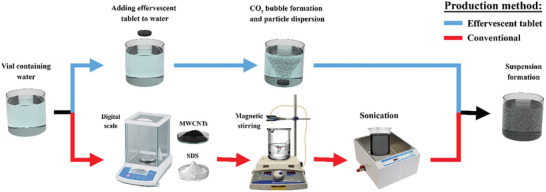
Nanofluid production processes. The top path shows the effervescent‐tablet approach, which includes adding a dry tablet to the liquid, with subsequent reaction and nanomaterial dispersion that forms the suspension. The bottom path shows the conventional route, which involves weighing the powder, initial mixing, and sonication to produce the dispersion.

The fabricated conventional suspensions were placed in the test‐rig liquid container and heated to 98 °C before being used as the working fluid in the test rig. **Figure** **6** shows the processes used to produce the effervescent‐tablet‐based and conventional nanofluids.

#### 2.6.2 Thermal Conductivity and Dispersion Stability

The thermal conductivities and dispersion stabilities of the suspensions were examined experimentally, with a hot‐wire device (THW‐L2, Thermtest) used to measure the thermal conductivity of each as‐prepared nanofluid, both conventional and effervescent‐tablet‐based, at a controlled temperature of 25 °C according to the ASTM D7896‐14 testing standard.^[^ ^57^ ^]^ To that end, an 80‐mL sample in a glass vial was placed on a thermoelectric dry bath (Thermtest) for temperature control, after which the hot‐wire probe was inserted and fully immersed in the suspension. Three measurements were taken 15 min apart (to ensure that previous measurements did not affect the thermal equilibrium and steady‐state conditions of the sample) with average values used. On the other hand, the dispersion stabilities of the as‐fabricated nanofluids were determined using two techniques, namely UV–vis spectroscopy and the sedimentation image capturing method.^[^ ^41^ ^]^ A UV‐2600 spectrophotometer (Shimadzu) was used to acquire UV‐vis spectra of the prepared suspensions in the 200–800 nm wavelength range, with a resolution of 1.0 nm. A Canon EOS 700D camera was employed to directly capture images after nanofluid production and on the 20th day that show changes in sample sedimentation behavior.

### 2.7 Oil‐Recovery Experiment

#### 2.7.1 Test‐Rig Set Up

**Figure 7:**
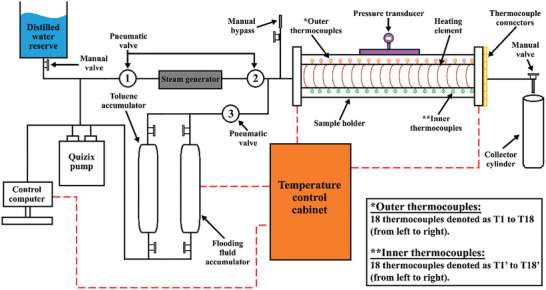
Schematic diagram of the experimental oil‐recovery‐system set up.

**Figure 8:**
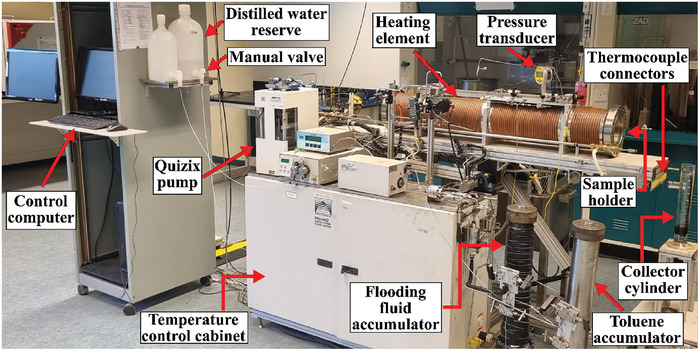
Photographic image of the test rig used in the oil‐recovery experiments.

A schematic and photographic image of the DSS‐945Z digital steamflood test‐rig system (Coretest Systems) used during various EOR processes is shown in **Figures** **7** and **8**, respectively. The test rig comprises three major components: the fluid injection system, fluid production system, and commercial Quizix (version 6.15, 2009) and Coretest (version 1.00.1807, 2012) software, with the former used to control and monitor the inserted fluid flow rate, and the latter used to control the core heating element and monitor changes in temperature throughout the core via 18 inner and 18 outer thermocouples, which can be performed manually or in semi‐automatic mode. It should also be noted that the heating element and outer thermocouples are placed along the length of the outer core tube to evenly distribute heat along the core content and measure any changes in tube temperature. The inner thermocouples were placed along the inner tube surface to measure temperature variations in the tube content. Moreover, the test‐rig setup enables steam injection, hot water flooding, or customized cyclic combinations within a packed sand core (diameter: 7.3 cm, length: 95 cm) under elevated or controlled pressure and temperature conditions. The test‐rig system can provide a maximum pore pressure of 1500 psi and maximum steam and flooded‐liquid temperatures of 325 and 98.5 °C, respectively. The test rig‐setup also contains a toluene accumulator that was used to clean the system at the end of each experiment. In addition, computer was used to control the pneumatic valves, which function through compressed air.

#### 2.7.2 Core Packing

Unconsolidated sand was multiply sifted to ensure uniform sand particles in the 0.15–1.18 mm range that were then used in the core‐packing process. The sand had a density of 2.143 g cm^−3^. Field data obtained from the Kuwait Lower Fares (LF) hydrocarbon formation revealed porosities of 30–40%; accordingly, a conservative value of 30% was selected. The data also revealed a water saturation of ≈20% and an oil saturation of ≈80%. Furthermore, a negligible gas saturation was assumed because heavy oil reservoirs are usually associated with low gas‐to‐oil ratios (GORs); therefore, their crude compositions are expected to be rather low in light hydrocarbon components, such as C1, C2, and C3.^[^ ^65^, ^66^, ^67^, ^68^, ^69^ ^]^ Moreover, because the bulk volume, pore volume, grain volume, oil volume, water volume, and sand mass need to be known for the sand‐packing process, they are calculated using Equations ((9), (10), (11), (12)):

(9) $$V_{p}=\phi V_{b}$$

(10) $$V_{\mathrm{grain}}=V_{p}-V_{b}$$

(11) $$V_{o}=S_{o}\times V_{p}$$

(12) $$V_{w}=S_{w}\times V_{p}$$

where V_p_, V_b_, V_grain_, V_o_, V_w_, S_o_, S_w_, and ϕ are the pore volume, bulk volume (which is equal to the core tube volume), grain volume, crude‐oil volume, water volume, crude‐oil saturation, water saturation, and porosity, respectively. Furthermore, the mass of the sand (m_sand_) was measured to be ≈6.5 kg. **Table** **4** summarizes data obtained from the abovementioned calculations and measurements.

**Table 4:** Summarizing key core‐packing parameters and their values.

Parameter	Units	Value
V_p_	cm^3^	1200
V_b_	cm^3^	4000
V_grain_	cm^3^	2800
V_o_	cm^3^	960
V_w_	cm^3^	240
S_o_	–	0.8
S_w_	–	0.2
Φ	–	0.3
m_sand_	kg	6.5

**Figure 9:**
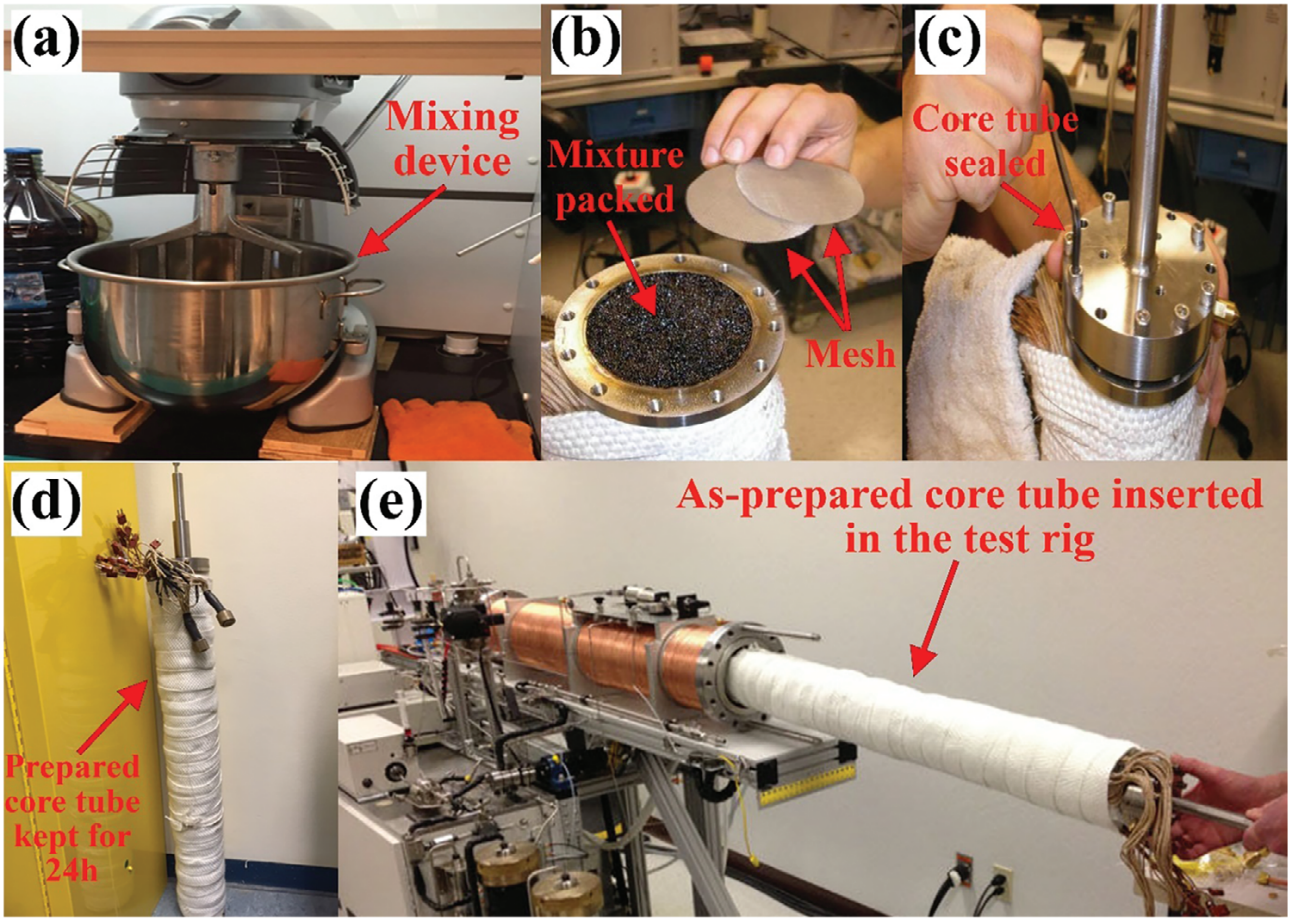
Sand, water, and crude‐oil packing process. a) Mixing device, b) packed mixture and meshes, c–e) core‐tube sealing, storage, and insertion into the test rig, respectively.

The specifications of the water and crude oil used in the mixtures are listed in Tables 1 and **5**, respectively. It should be noted that the crude oil was obtained from an oil well in the LF formation, whereas the water was prepared in the laboratory according to the characteristics of the actual water in the LF formation, as detailed in Section 2.1. In addition, the hydrocarbons (i.e., C1–C32+) in the as‐received crude oil were determined using gas chromatography (GC 7890A, Agilent Technologies). Furthermore, oil, water, and sand were manually mixed, and the mixture was preheated to 50 °C to increase its mobility. The three components were subsequently homogenized using a mixing device (**Figure** **9**a) for 3 h to ensure consistency. The mixture was then slowly added to the core tube (Figure 9b) and placed on a holder with a vibrating surface to assist displacement of the mixture. A fine mesh was added to the bottom end of the core tube to prevent sand particles from separating. A metal rod was used to push the sand mixture into the core tube to compress it uniformly. Once the packing process was complete, a second mesh was placed at the top end of the core tube prior to being sealed (Figure 9b,c). The sealed core tube was maintained vertically for 24 h to exploit gravity during the packing process (Figure 9d) before being inserted into the test rig for EOR testing (Figure 9e).

**Table 5:** Measured properties and composition of the as‐received crude oil.

Measurement	Units	Value
Property		
API	°	11.90
Specific gravity at 15 °C	–	0.98673
Density at 15 °C	g cm^−3^	0.98576
Density at 25 °C	g cm^−3^	0.97953
Kinematic viscosity at 25 °C	mm^2^ s^−1^	2590
Component		
C1–C5	wt%	0.00
C6–C10	wt%	2.09
C11–C15	wt%	3.22
C16–C20	wt%	4.47
C21–C25	wt%	4.50
C26–C30	wt%	4.03
C31–C32	wt%	2.24
C32+	wt%	79.44

#### 2.7.3 Enhanced Oil Recovery Testing

EOR was examined using 13 scenarios, which included a single conventional‐fluid stage, two conventional‐fluid stages, and two conventional and nanofluids stages; these scenarios are summarized in **Table** **6**. It is important to note that 400 cm^3^ of steam was initially injected until the first drop of oil was obtained when steam injection was combined with other flooding processes, which was followed by the introduction of a second recovery approach involving 800 cm^3^ of water or a nanofluid, leading to a total equal to the pore volume of the core (i.e., 1200 cm^3^). In addition, the steam‐injection and liquid (or nanofluid) flooding rates were selected to be 10 and 5 cm^3^ min^−1^, respectively, to maintain an inner core pressure of 250 psi. Furthermore, the core temperature throughout the oil recovery experiments was fixed at 30 °C. The abovementioned pressures and temperatures were maintained to reflect the in‐situ reservoir conditions. One of the 13 scenarios was employed in each EOR experiment, after which the extracted oil was collected and analyzed.

**Table 6:** Data for the working fluids used in the 13 oil‐recovery scenarios.

Scenario no.	Working fluid details							
	Type		Volume [cm^3^]		Temperature [°C]		Flow rate [cm^3^ min^−1^]	
	Stage I	Stage II	Stage I	Stage II	Stage I	Stage II	Stage I	Stage II
1	Pure steam	–	1200	–	150	–	10	–
2	Treated seawater	–	1200	–	98	–	5	–
3	Distilled water	–	1200	–	98	–	5	–
4	Steam	Treated seawater	400	800	150	98	10	5
5	Steam	Distilled water	400	800	150	98	10	5
6	Steam	Case 1 tablet‐base nanofluid	400	800	150	98	10	5
7	Steam	Case 2 tablet‐base nanofluid	400	800	150	98	10	5
8	Steam	Case 3 tablet‐base nanofluid	400	800	150	98	10	5
9	Steam	Case 4 tablet‐base nanofluid	400	800	150	98	10	5
10	Steam	Case 5 tablet‐base nanofluid	400	800	150	98	10	5
11	Steam	Conventional nanofluid (0.01 vol%)	400	800	150	98	10	5
12	Steam	Conventional nanofluid (0.05 vol%)	400	800	150	98	10	5
13	Steam	Conventional nanofluid (0.10 vol%)	400	800	150	98	10	5

## 3 Results and Discussion

### 3.1 X‐Ray Diffractometry

**Figure 10:**
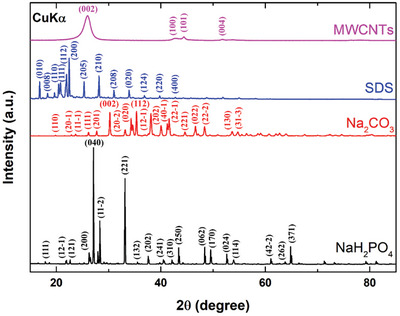
XRD patterns of the powder feedstocks.

Each as‐received powder used as feedstock for the formation of the effervescent‐tablets was subjected to X‐ray diffractometry. The diffraction pattern acquired for each examined sample provides information of the material type by comparison with the device database patterns. **Figure** **10** shows XRD patterns acquired for all four powders, which were obtained through electromagnetic beam reflection from the crystalline planes of the particles within the examined samples. Furthermore, the electromagnetic beam itself is formed by emissions generated from the CuKα X‐ray source of the device.

The acquired XRD patterns are indicative of the crystalline structures of the examined specimens. The as‐received powders used as feedstock were confirmed as MWCNTs (PDF# 00‐058‐1638), SDS (PDF# 00‐039‐1996), NaH_2_PO_4_ (PDF# 01‐084‐0112), and Na_2_CO_3_ (PDF# 05‐001‐0004) by comparing the acquired experimental data with the patterns available in the device database; these results are also in good agreement with the literature.^[^ ^70^, ^71^, ^72^, ^73^ ^]^ The crystallite sizes of the MWCNT, SDS, NaH_2_PO_4_, and Na_2_CO_3_ samples were calculated using the Scherrer formula (Equation (13)):^[^ ^74^, ^75^, ^76^, ^77^ ^]^

(13) $$D_{hkl}=\frac{F\lambda }{\beta_{hkl}cos\theta_{hkl}}$$

where *F* is the shape factor and has a value of ≈0.90, λ is the wavelength of the radiation source (i.e., CuK_α_) and has a value of ≈0.154 nm, β_*hkl*_ is the full width at half the maximum of the (*hkl*) diffraction peak, and θ_*hkl*_ is the Bragg angle of the (*hkl*) peak. As such, the MWCNTs, SDS, NaH_2_PO_4_, and Na_2_CO_3_ were found to have average crystallite sizes of 8.3, 47.7, 33.9, and 83.9 nm, respectively. Furthermore, the 0 0 2 (MWCNTs), 2 0 0 (SDS), 0 4 0 (NaH_2_PO_4_), and 1 1 2 (Na_2_CO_3_) planes were observed to exhibit the most‐intense peaks in the XRD patterns of the samples.

### 3.2 FE‐SEM and EDS

**Figure 11:**
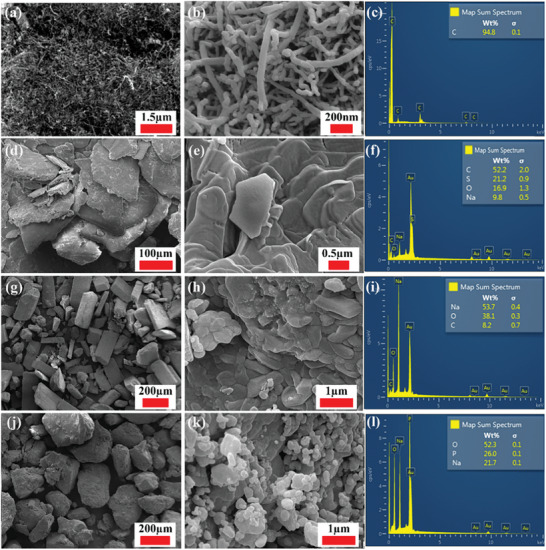
FE‐SEM images and EDS spectra of a–c) the MWCNTs, d–f) SDS, g–i) Na_2_CO_3_, and j–l) and NaH_2_PO_4_ powders.

**Figure** **11** shows FE‐SEM images and EDS data for the as‐obtained MWCNT, SDS, Na_2_CO_3_, and NaH_2_PO_4_ powders. The FE‐SEM image shown in Figure 11b distinctly reveals tube‐like structures, consistent with the anticipated morphology of the CNTs; these tubes clearly exhibited a notable tendency to agglomerate, which is common behavior observed for such nanomaterials and is attributable to the high surface energies of the MWCNTs that arise from their high surface‐to‐volume ratios.^[^ ^78^ ^]^ Such characteristics lead to mutual inter‐CNT attraction that thermodynamically stabilizes the nanomaterial. Additionally, the tube‐shaped nanomaterials have outer diameters that range between ≈26 and 48 nm. In contrast, the SDS particles shown in Figure 11d,e lack a consistent morphological structure and form highly agglomerated, nonuniform flake‐like structures that are ≈268–934 nm in size. Moreover, bulk Na_2_CO_3_ exhibits a rod‐like morphology that also consists of small semi‐spherical (or roughly spherical) particles, as shown in Figure 11g,h. NaH_2_PO_4_ exhibited a more random but close‐to‐spherical bulk structure (Figure 11j); however, each bulk material is composed of an array of spherical particle clusters (Figure 11k). Notably, Na_2_CO_3_ exhibited a higher level of particle aggregation than NaH_2_PO_4_, as shown in Figure 11h,k. Such particle aggregation resulted in the formation of microscale bulk materials, with sizes of ≈38.6–274 µm for Na_2_CO_3_ and 17.4–342.6 µm for NaH_2_PO_4_. The small particles observed on the surfaces of Na_2_CO_3_ and NaH_2_PO_4_ (Figure 11h,k) are possibly ascribable to interface nucleation, as described by Mahendiran et al.^[^ ^79^ ^]^

In contrast, the EDS data for the as‐obtained powders are shown in Figure 11c,f,i,l). The MWCNT sample contains 94.8 wt% carbon (Figure 11c), which is in acceptable agreement with the manufacturer's data (i.e., purity > 95 wt%). Furthermore, the EDS powder spectrum of SDS in Figure 11f shows C, S, O, and Na at levels of 52.2, 21.2, 16.9, and 9.8 wt%, respectively; the S in the analyzed sample is mainly attributable to unavoidable exposure to the surrounding atmosphere during insulation in the device chamber. The Na_2_CO_3_ sample contains 53.7 wt% Na, 38.1 wt% O, and 8.2 wt% C, whereas NaH_2_PO_4_ contains 52.3 wt% O, 26 wt% P, and 21.7 wt% Na. Based on the four EDS powder spectra, it was concluded that the samples are consistent with supplier specifications, with contamination only observed for the SDS sample. It should be noted that the peaks corresponding to elemental gold in the EDS spectra (i.e., in Figure 11f,(i),(l)), although excluded from the elemental calculations, is ascribable to the layer coated on each sample prior to being introduced into the characterization device; samples were coated to increase conductivity and improve the overall analysis outcomes as a consequence.

### 3.3 Thermodynamic Analyses of the Effervescent‐Tablet‐Based Nanofluids

**Figure 12:**
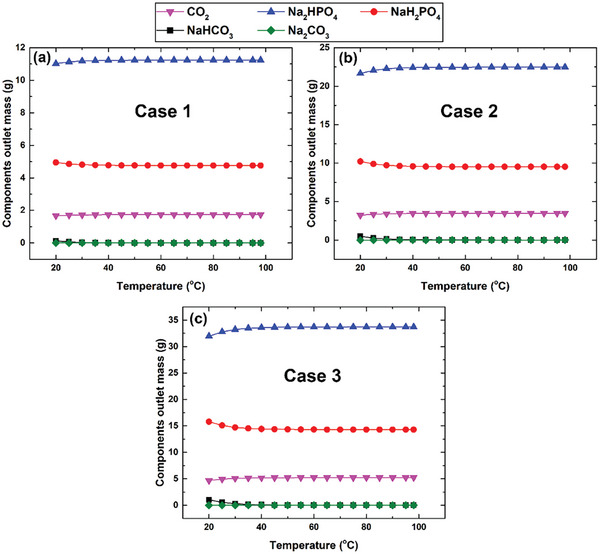
Component masses present in the effervescent solutions as functions of Case and temperature.

**Figure 13:**
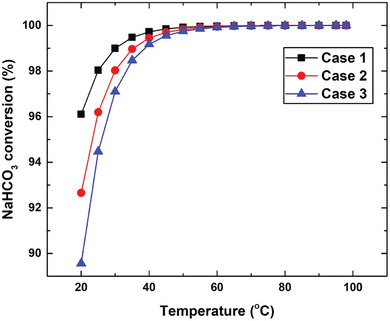
NaHCO_3_ conversion as functions of temperatures and studied case.

The amounts of components comprising the effervescent solution at equilibrium for the various cases and in the temperature range mentioned in Section 2.5 are shown in **Figure** **12**. The concentrations of the effervescent agents (Na_2_CO_3_ and NaH_2_PO_4_) were directly proportional to the amount of CO_2_ generated via the reactions described by Equations (2) and (3). Cases 3–5 generated a maximum of 4.9 g of CO_2_ because the masses of the effervescent agents used in these cases are the same as those used in the other cases (i.e., Cases 1 and 2). In addition, the results in Figure 12 show that no Na_2_CO_3_ is present in the solution at any temperature range or case examined, which reveals that Na_2_CO_3_ was completely converted via Equation (2) at all temperatures examined. This observation is attributable to the negative change in the Gibbs free energy associated with Equation (2) (i.e., −6.9 kJ mol^−1^ at 25 °C).^[^ ^39^ ^]^ Additionally, the heat of the reaction of Equation (2) is −30 kJ mol^−1^ under standard conditions,^[^ ^80^ ^]^ which means that the reaction is exothermic and equilibrium is favored at low temperatures. On the other hand, the equilibrium mass of NaHCO_3_, NaH_2_PO_4_, Na_2_HPO_4_, and CO_2_ remained constant, at 0.005, 4.8, 11.2, and 1.7 g, respectively for Case 1 at ≥45 °C. However, the masses of these components do not vary at ≥55 °C for Cases 2 and 3. For example, the masses of NaHCO_3_, NaH_2_PO_4_, Na_2_HPO_4_, and CO_2_ are ≈0.004, 9.5, 22.5, and 3.5 g, respectively, in Case 2. Therefore, it was concluded that complete conversion through Equation (3) is achieved at 45 °C for Case 1, whereas a temperature of 55 °C is required for Cases 2 and 3 to achieve full conversion, as illustrated in **Figure** **13**, which reveals that complete conversion is not thermodynamically achievable at room temperature owing to the positive value of the standard Gibbs free energy and the endothermic nature of the reaction depicted in Equation (3), as reported previously.^[^ ^39^ ^]^ In addition, most sodium bicarbonate (98%) was converted at room temperature in Case 1, whereas Case 3 exhibited the lowest conversion (94.5%), as shown in Figure 13, which can be explained by investigating the equilibrium concentrations of the species involved in the reactions, as presented in Equations (2) and (3). The mass of NaH_2_PO_4_ is higher in Case 3 than in Cases 1 and 2. Hence, because the same volume of water was used to dissolve the tablets in all examined cases, Case 3 exhibited the highest concentration of NaH_2_PO_4_, as reflected by Equation (5). In addition, the reaction shown in Equation (2), which undergoes complete conversion, explains why more Na_2_HPO_4_ is generated in Case 3 than in Cases 2 and 1. It should be noted that Na_2_HPO_4_ is generated according to the reaction shown in Equation 3; therefore, the concentration of this chemical compound in solution is higher for Case 3 than for Cases 1 and 2 at room temperature. For instance, the concentrations of Na_2_HPO_4_ in Cases 3 and 1 at room temperature are 0.024 and 0.008 g L^−1^, respectively.

According to Le Chatelier's principle,^[^ ^81^ ^]^ the reaction depicted in Equation (3) will shift toward the left to a greater extent in Case 3 compared to the other two preceding cases. Consequently, a higher amount of NaHCO_3_ is regenerated from the components used in Case 3. As such, the least amount of NaHCO_3_ (denoted by Equation (6)) was converted. Following this principle, Case 1 exhibited the lowest concentration of NaH_2_PO_4_; consequently, the smallest amount of NaHCO_3_ is regenerated via Equation (3) in this case; therefore, it exhibited the highest conversion at room temperature, as shown in Figure 13. However, the equilibrium is significantly influenced by the reaction temperature above 45 °C owing to the endothermic nature of the reaction in Equation (3), which exerts a greater influence than substance concentration. Hence, the reaction shifts toward the production of CO_2_, and complete conversion is achieved.

**Figure 14:**
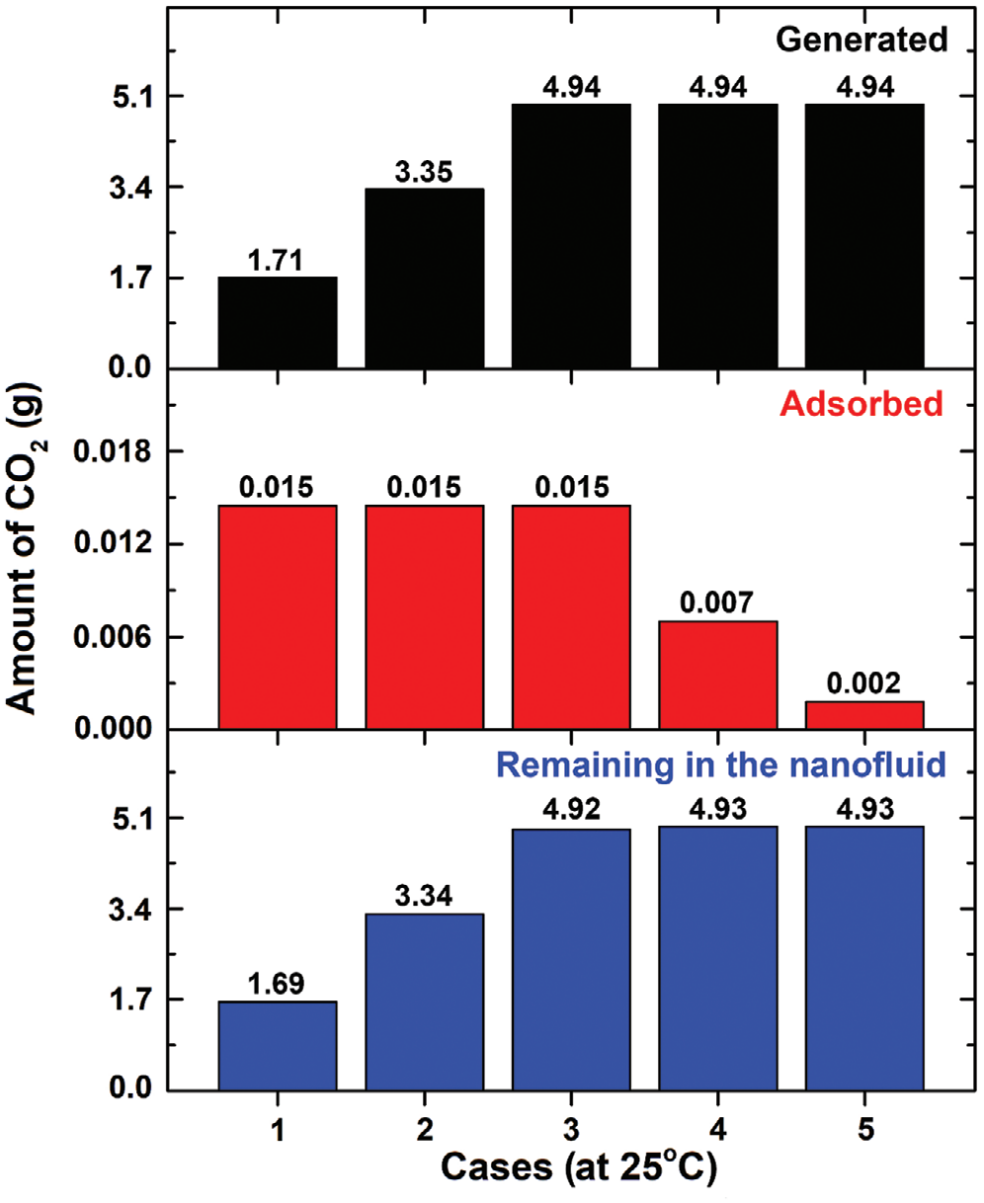
Amounts of CO_2_ generated, adsorbed by the MWCNT, and remaining in the nanofluid for the five investigated cases.

On the other hand, **Figure** **14** shows the amounts of CO_2_ adsorbed by the MWCNT nanomaterial at 25 °C for the cases listed in Table 3, with Cases 1–3 adsorbing the most CO_2_ among the five cases with different effervescent‐tablet contents. This observation is ascribable to the higher quantities of MWCNT present in the tablets in these three cases (i.e., 2.098 g) compared with the other two cases (i.e., 1.055 and 0.211 g for Cases 4 and 5, respectively). Therefore, Cases 1–3 adsorb the same amount of CO_2_ (0.015 g). In addition, Figure 14 shows that the remaining generated CO_2_ (4.93 g) is the highest in the nanofluid produced in Case 5, whereas Case 1 exhibited the lowest (1.69 g). As discussed earlier, Cases 3–5 generate the most CO_2_ because they contain the most effervescent agents. However, Case 5 contains the lowest amount of MWCNT leading to the lowest amount of adsorbed CO_2_ among the studied cases, as shown in Figure 14. Accordingly, the aforementioned case was expected to exhibit the highest amount of remaining CO_2_ when the nanofluid was physically fabricated. In contrast, Case 1 was predicted to adsorb the highest amount of generated CO_2_ leading to the lowest amount of CO_2_ present in the suspension.

### 3.4 Thermal Properties of the Dispersion

**Figure 15:**
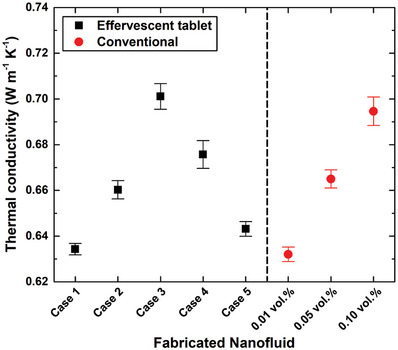
Thermal conductivities of as‐fabricated suspensions. Cases 1–5 were produced using effervescent‐tablets, and the 0.01–0.10 vol% nanofluids were conventionally fabricated.

The thermal conductivities of the nanofluids, both effervescent‐tablet‐based and conventional, were measured following preparation, the results of which are shown in **Figure** **15**. Furthermore, the effervescent‐tablet cases (i.e., Cases 1–5) shown in Figure 15 correspond to those previously mentioned in Table 3, whereas the volume percentages show the concentrations of various conventional nanofluid. Inspection of Cases 1–5 reveals that the use of the effervescent agent to mix the dispersed MWCNTs plays a primary role in enhancing the thermal conductivity of the as‐prepared suspension, which becomes clear when the three cases with equivalent MWCNT concentrations are compared, despite twice and three‐times the amounts of effervescent agent used in Cases 2 and 3, respectively, compared to Case 1. Therefore, thermal conductivities of 0.701, 0.660, and 0.634 W m^−1^ K^−1^ were obtained for Cases 3, 2, and 1, respectively; these values represent enhancements of 15.68% (Case 3), 8.91% (Case 2), and 4.62% (Case 1) over that of the base fluid. In contrast, reducing the MWCNT amount from 2.098 to 0.211 g while maintaining the same amount of effervescent agent as that used in Case 3 (i.e., the highest mass of effervescent agent) led to inferior thermal properties, which is commonly observed in the most physically stable and homogenously dispersed suspensions, in which increasing the concentration of dispersed material results in an increase in thermal conductivity of the nanofluid, and vice versa.^[^ ^82^, ^83^ ^]^ Accordingly, thermal conductivities of 0.676 and 0.643 W m^−1^ K^−1^ were determined for Cases 4 and 5, respectively; these values are 11.55% and 6.11% higher, respectively, than those of the base fluid. Values of 0.632, 0.665, and 0.695 W m^−1^ K^−1^ were measured for the 0.01 0.05, and 0.10 vol% conventional‐nanofluid suspensions, respectively. It should be noted that factors other than mixing and nanomaterial concentration can also influence the thermal conductivity of the suspension, including the presence of a surfactant and the fabrication temperature of the as‐prepared nanofluid.^[^ ^84^, ^85^ ^]^ However, the surfactant affects the properties of a nanofluid less significantly than the other two factors (i.e., mixing and nanomaterial concentration), and the suspension temperature is easily controlled during the production phase to ensure that it does not affect thermal conductivity.^[^ ^86^, ^87^, ^88^, ^89^ ^]^

### 3.5 Nanofluid Physical Stability

**Figure 16:**
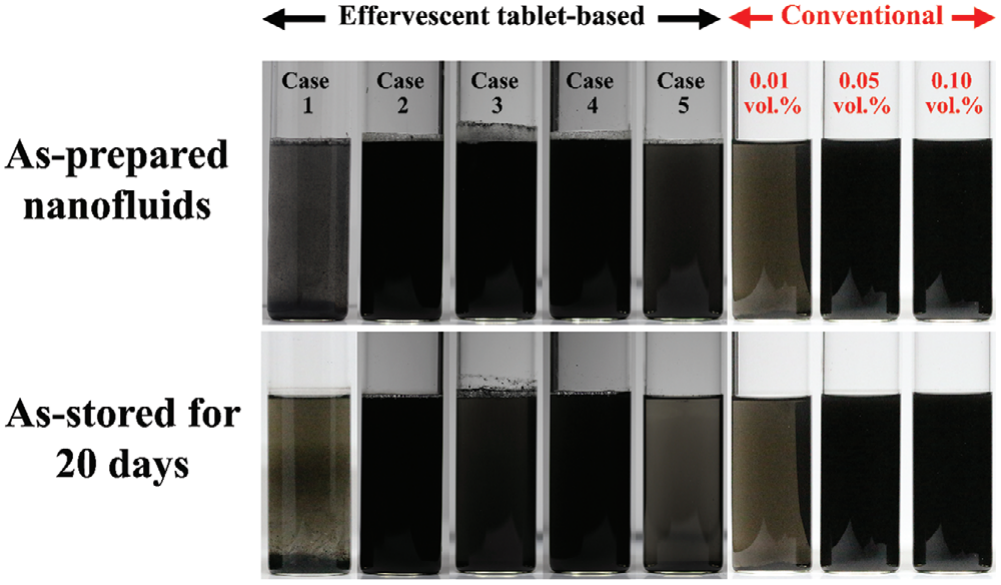
Visual images of various nanofluids following preparation and on the 20th day. Cases 1–5 were produced using the effervescent‐tablet approach, while the 0.01–0.10 vol% nanofluids were fabricated using the conventional route.

**Figure 17:**
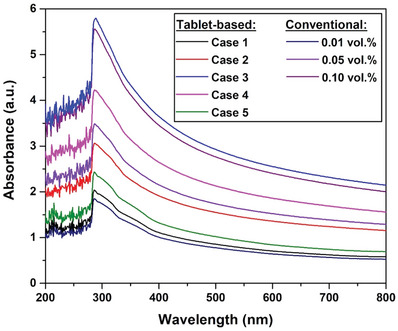
UV–vis analysis of as‐fabricated suspensions. Cases 1–5 were prepared using the effervescent‐tablet method, while the 0.01–0.10 vol% nanofluids were produced using the conventional approach.

The stability of the dispersed nanomaterial within the base fluid for each category of nanofluid (i.e., effervescent‐tablet‐based and conventional) was analyzed using the photographic image capturing technique and UV–vis spectroscopy, the results of which are shown in **Figures** **16** and **17**. Visual inspection of the effervescent‐tablet‐based nanofluids (Figure 16) reveals that Cases 2, 3, and 4 are darker in color than Cases 1 and 5 after the suspension‐preparation stage. Such variations in nanofluid color do not necessarily reflect dispersed‐particle stability within the host, as it may be due to the MWCNT concentration used to prepare each sample. However, Case 1 is evidently less stable than its counterparts when nanofluids with similar MWCNT concentrations (i.e., Cases 1–3) are compared. Moreover, a noticeable amount of sediment was observed in Case 1, which is attributable to the dispersed nanomaterial agglomerating into larger clusters; such clusters are heavier than the individual nanomaterial particles, which generates a higher drag force as a consequence. The MWCNT clusters tend to settle at the bottom as a sediment once the drag force of the agglomerated nanomaterial exceeds the buoyancy force provided by the base fluid, as shown in Figure 16 (Case 1). This observation supports the previous notion that the nanofluid in Case 1 is less stable than those in Cases 2 and 3. In addition, the images obtained on the 20th day clearly confirm the abovementioned dispersion‐stability conclusion. On the other hand, while conventional nanofluid suspensions with concentrations of 0.10 and 0.05 vol% appear to be physically stable, traces of sediment were observed in the 0.01 vol% sample. In addition, no significant changes in dispersion stability were observed, even after 20 days of sample storage. Moreover, a comparison of the 0.01 vol% suspension with the Case 5 nanofluid, which shares the same MWCNT concentration, reveals that the latter is more physically stable than the former following the preparation phase owing to its darker color. In contrast, the 0.01 vol% sample exhibited better long‐term stability than the Case 5 suspension on the 20th day. It should be noted that the foam observed in the Cases‐1–5 samples (Figure 16) is formed through the reactions of the effervescent agents in water; such a layer was not observed for the conventional nanofluid samples.

In contrast, the UV–vis results compare the levels of dispersion stability of nanofluid samples with the same MWCNT concentration, as shown in Figure **17**, which includes effervescent‐tablet Cases 1–3 and the 0.10 vol% conventional nanofluid, effervescent‐tablet Case 4 and the 0.05 vol% conventional nanofluid, and effervescent‐tablet Case 5 and the 0.01 vol% conventional nanofluid. The suspension in Case 3 displayed the highest dispersed‐particle stability, followed by the 0.10 vol% conventional fluid, Case 2, and Case 1 (lowest) respectively. Although visually determining the levels of dispersion stability for Cases 2–3 and the 0.10 vol% conventional fluid was not possible (Figure 16), the visual image of the Case 1 suspension is clearly in good agreement with the UV–vis data (i.e., the least stable among samples with similar MWCNT concentrations). Furthermore, the Case 4 suspension was observed to be more stable than the 0.05 vol% conventional nanofluid, while Case 5 exhibited a higher level of physical stability than the 0.01 vol% conventional fluid. The visual images in Figure 16 do not show differences in dispersed‐particle stability between the Case 4 and 0.05 vol% conventional nanofluids, consistent with the UV–vis results obtained for the suspensions fabricated using the effervescent‐tablets in Case 5 and the 0.01 vol% conventional nanofluid, in which traces of sediment are clearly visible in the conventional sample along with its lighter apparent color compared to that of the Case 5 sample. From the previous observation, it can be concluded that the 1:1:20.4:6 wight ratio is the optimum mixture concentration for forming a stable effervescent‐tablet‐based suspension.

### 3.6 Oil‐Recovery Analysis

**Figure 18:**
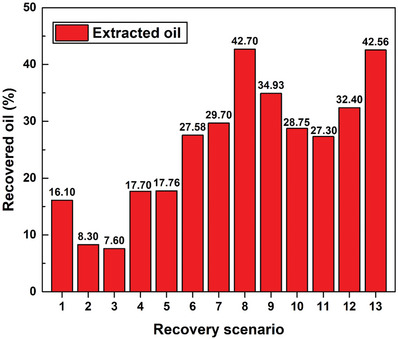
Percentages of extracted oil obtained using various oil‐recovery scenarios.

**Figure 19:**
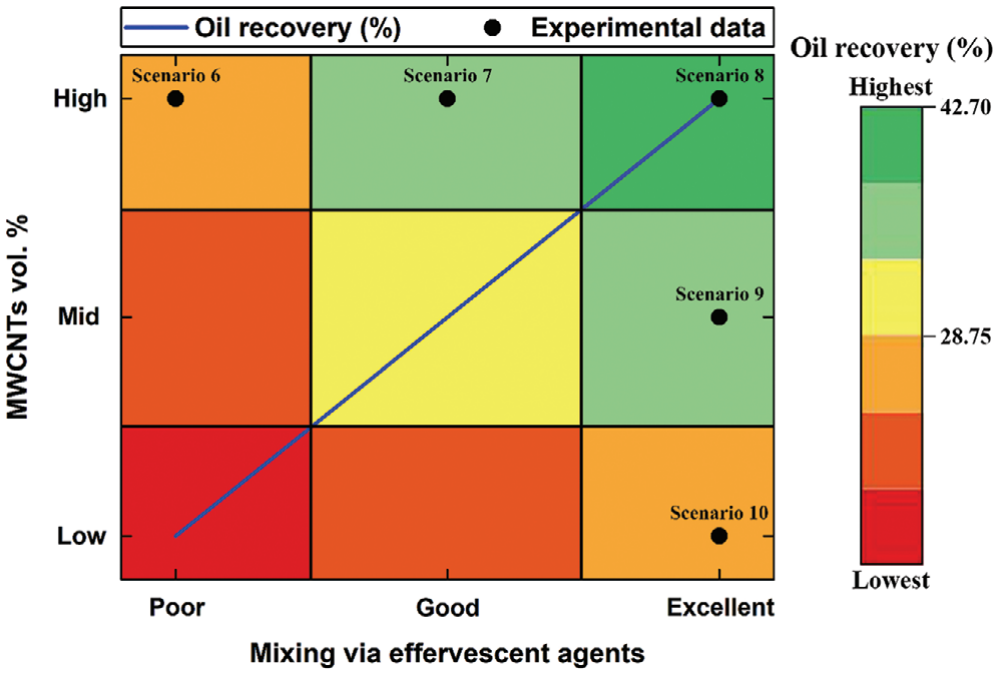
Influence of MWCNT concentration and its mixing efficiency on the percentage of recovered oil, where green and red correspond to the highest and lowest oil recoveries, respectively.

Oil‐recovery experiments were conducted for all 13 scenarios using the test rig, the details of which are provided in Table 6, with the corresponding results shown in **Figure** **18**. It should be noted that the numbers shown on the x‐axis in Figure 18 correspond to the scenarios listed in Table 6 (i.e., “1” corresponds to Scenario 1, “2” to Scenario 2, etc.). Furthermore, among the conventional single‐stage recovery approaches (i.e., Scenarios 1–3), pure steam injection (Scenario 1) delivered more extracted oil than treated seawater flooding (Scenario 2) and DW flooding (Scenario 3). More precisely, 16.10% of the initial crude oil contained in the sample in the test rig was extracted using the recovery method in Scenario 1, which is 93.98% and 111.84% higher than the amounts of oil extracted employing Scenarios 2 and 3, respectively. These outcomes are likely ascribable to the higher thermal impact of steam on the thermophysical properties of the oil compared to the two other liquid flooding processes, especially taking into consideration that the steam was injected at 150 °C compared to the 98 °C used for both flooding liquids. As for the two‐stage conventional recovery route that includes steam injection (stage 1) and hot water flooding (stage 2), Scenarios 4 and 5 exhibited very similar recovery results, with a variation of only 0.34% observed. On the other hand, the results obtained using the advanced two‐stage recovery method, which consists of steam injection and nanofluid flooding, can be divided into two groups based on the suspension‐production approach used. The first group includes effervescent‐tablet‐based nanofluids (Scenarios 6–10), whereas the second group includes conventional suspensions (Scenarios 11–13). Inspection of the results obtained for the first group reveals that the suspension with highest MWCNT and effervescent‐agent concentrations (i.e., scenario 8) provided optimum oil recovery among Scenarios 6–10. Specifically, 27.58%, 29.70%, 42.70%, 34.93%, and 28.75%, of the oil was extracted in scenarios 6–10, respectively. Furthermore, the dispersion (or mixing) efficiency of the nanomaterials that form the suspensions, along with the concentrations of added material, play crucial roles in determining the oil‐extraction outcome. For example, Scenarios 6–8 used identical MWCNT concentrations but different effervescent‐agent concentrations; as such, Scenario 8 (highest effervescent‐agent concentration) extracted 54.82% and 43.77% more oil than Scenarios 7 (mid‐effervescent‐agent concentration) and 6 (lowest effervescent‐agent concentration), respectively. Additionally, varying the MWCNT concentration while fixing the effervescent agent concentration also notably influenced the amount of recovered crude oil, as observed for Scenarios 8–10. Specifically, the recovery recorded for Scenario 8 (highest MWCNT concentration) is 22.24% and 48.52% higher than those of Scenarios 9 (mid‐MWCNT concentration) and 10 (lowest MWCNT concentration), respectively. Based on the abovementioned two effervescent‐tablet‐based nanofluid examples, which involved fixing one factor and varying the nanomaterial or effervescent agent concentration, an approximate oil‐recovery relationship involving the two aforementioned factors shown in **Figure** **19** was derived. The derived relationship is based on the behavior of the obtained experimental data and that expected from other scenarios that were not included in this study. The oil recovered in group two (i.e., Scenarios 11–13) was found to be mainly impacted by the concentration of dispersed MWCNT. The 0.10 vol% suspension (Scenario 13) afforded an oil recovery of 42.56%, which is higher than the amount of oil extracted via Scenarios 12 (0.05 vol% suspension) and 11 (0.01 vol% suspension) by 31.36% and 55.90%, respectively. However, comparing the optimally mixed effervescent‐tablet‐based dispersions with conventional nanofluids with similar MWCNT concentrations, namely, Scenario 8 versus 13, 9 versus 12, and 10 versus 11, clearly reveals small differences (<10%) in the amounts of recovered oil when the same nanomaterial concentrations are used, with recovered‐oil differences of 0.33%, 7.52%, and 5.17% recorded, respectively. These findings reveal that the use of a nanofluid as the flooding fluid enhances the oil‐recovery process, irrespective of the suspension‐production approach used. Nevertheless, using effervescent‐tablets to fabricate nanofluids in an actual oil field does not require further modifications to existing drilling rigs or the infrastructure, which is not the case for the conventionally made suspensions.

### 3.7 Properties and Composition of the Extracted Oil

**Figure 20:**
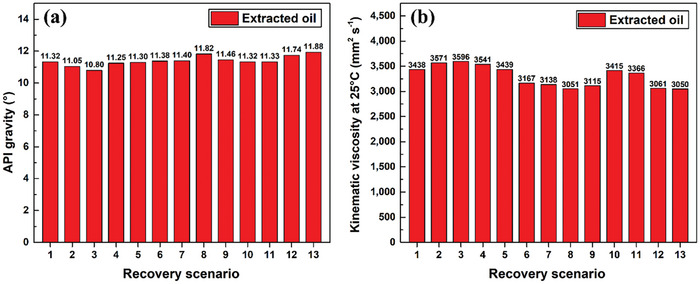
Properties of as‐extracted oil samples obtained via various recovery scenarios: a) API gravity and b) kinematic viscosity at 25 °C.

The API gravity, kinematic viscosity, specific gravity, and composition of the oil extracted in each recovery scenario were determined. In terms of the API gravity of the extracted oil, all recovery scenarios provided oil with lower API gravities than that of the initial crude oil (i.e., 11.90°) used to core‐pack the test rig. **Figure** **20**a shows the API gravity of the oil extracted in each recovery scenario. Such lower API gravities indicate that the recovered oils are heavier than the original. Furthermore, among the conventional single‐stage recovery protocols (i.e., Scenarios 1–3), steam injection provided the lightest extractant, with an API gravity of 11.32°, which is to be compared with that obtained using treated seawater (API gravity: 11.05°) and distilled water (API gravity: 10.80°) flooding (i.e., Scenarios 2 and 3). Moreover, the API gravity of the recovered oil was found to be close to that obtained by pure steam injection when steam injection was combined with seawater and distilled water flooding using two‐stage recovery approaches (i.e., Scenarios 4 and 5), with reductions of 0.62% and 0.18% recorded for Scenarios 4 and 5, respectively. The highest API gravities were obtained for the advanced recovery scenarios that use nanofluids in their recovery process (i.e., Scenarios 6–13), with Scenarios 8 (effervescent‐tablet) and 13 (conventional) delivering API gravities of 11.82° and 11.88°, respectively. The API gravity of the extracted oil was observed to decrease with decreasing MWCNT concentration in the employed well‐dispersed nanofluid, as evidenced in Scenarios 8–10 and 13–11. It is believed that this outcome is ascribable to differences in the thermal effectiveness of each flooding suspension. Therefore, the thermal effectiveness of the nanofluid used in the recovery process increases with increasing dispersed‐nanomaterial concentration, and vice versa. However, the oil extracted in all recovery scenarios remained in the “extra heavy crude oil” category because the as‐obtained API gravities lie in the 10–20° range.^[^ ^17^, ^18^, ^19^ ^]^

The kinematic viscosities of the oil extracted in the various scenarios exhibit an inverse relationship with API gravity, as shown in Figure 20b. This observation is ascribable to a higher API gravity corresponding to lighter crude oil that has a lower kinematic viscosity as a consequence, and vice versa.^[^ ^90^, ^91^ ^]^ Furthermore, analysis of the oils obtained using the various conventional recovery approaches (i.e., Scenarios 1–5) revealed that the oil obtained via Scenario 1 exhibited the lowest kinematic viscosity of 3438 mm^2^ s^−1^, whereas Scenario 3 led to the highest kinematic viscosity of 3596 mm^2^ s^−1^; these two values differ only slightly (i.e., by 4.49%). Among the advanced recovery process (i.e., Scenarios 6–13), the properties of the extracted crude obtained via Scenarios 13 and 10 exhibited the lowest and highest kinematic viscosities, respectively, which differed by 11.29%; hence, the two recovery scenarios led to notably different properties. It should be noted that Scenarios 13 (conventional nanofluid) and 8 (effervescent‐tablet‐based suspension) have similar kinematic viscosities that differ by only 0.03%. Overall, the oil recovered via Scenarios 13 and 3 exhibited the lowest and highest kinematic viscosities, respectively, among the examined recovery scenarios. Understanding how kinematic viscosity is related to the recovery scenario is crucial, as this knowledge is expected to profoundly influence the future management of crude oil. This becomes particularly important when considering the pumping process because a higher‐viscosity oil demands more pump power and vice versa. Therefore, recognizing these viscosity relationships is vital for future efficient and optimal crude‐oil handling.

**Figure 21:**
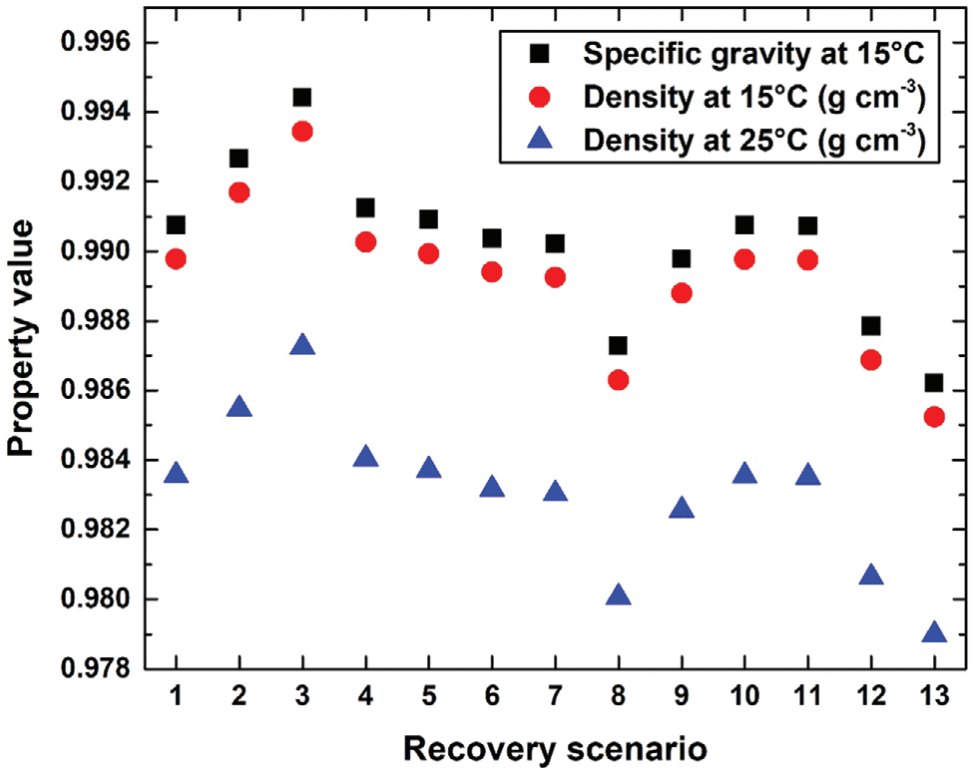
Relationships between recovery scenario and the specific gravity and density of the extracted crude oil.

**Figure** **21** shows other measured properties, including specific gravity and density at both 15 and 25 °C. The oil extracted via different recovery scenarios exhibited specific gravities of 0.986–0.994 at 15 °C and densities of 0.985–0.993and 0.979–0.987 g cm^−3^ at 15 and 25 °C, respectively. The highest values of the three aforementioned properties were observed for the crude oil extracted using Scenario 3, whereas the lowest values were obtained using Scenario 13. Furthermore, the crude obtained via Scenario 8 exhibited similar values for these three properties to those acquired through Scenario 13, with a difference of only ≈0.11%.

The compositions of the oil recovered via the various scenarios were determined and are listed in **Table** **7**. Knowing the composition of the oil extracted in each recovery scenario is crucial for several reasons, but mainly because it influences the price of the crude oil and its refining process.^[^ ^92^, ^93^ ^]^ Table 7 reveals that the recovery scenarios involving nanofluids fabricated using effervescent‐tablets containing the largest amount of effervescent agents (i.e., Scenario 8–10) resulted in the present of the C1–C5 components in the extracted oil, which suggests that the crude material underwent thermal cracking during recovery and that the CO_2_ generated from the effervescent‐tablets, which is a miscible gas associated with the nanofluid‐fabrication process, facilitates better hydrocarbon displacement and mobilization during the recovery process. The latter is likely to be the most‐plausible reason, given that the oil obtained via Scenario 10 contains more C1–C5 (6.29 wt%) than that from Scenario 8 (1.40 wt%). Moreover, knowing that MWCNTs tend to adsorb CO_2_,^[^ ^94^, ^95^ ^]^ increasing their concentration is likely to result in less free CO_2_ within the flood, and vice versa. Alternatively, this observation may also be ascribable to the effervescent‐tablet scenarios that have the potential to create a trihybrid EOR production scheme, where thermal, chemical, and miscible flooding are involved owing to the use of a high‐temperature flood, nanofluid, and CO_2_ gas (in the form of bubbles). Heavier components (i.e., C32+) were found to be highly present in the oils obtained using conventional recovery methods (i.e., Scenarios 1–5). Additionally, the same components exhibit proportional relationships with the dispersed nanomaterial concentration when an advanced recovery method is used. Increasing the MWCNT concentration in the flooding nanofluid increased the percentage of C32+ components in the as‐recovered crude, as was observed for the oil recovered via Scenarios 6–13.

**Table 7:** Composition of the as‐extracted oil obtained from each recovery scenario.

Experimental scenario	Component [wt%]							
	C1–C5	C6–C10	C11–C15	C16–C20	C21–C25	C26–C30	C31–C32	C32+
1	0.00	0.15	2.87	4.18	4.48	4.35	2.02	81.95
2	0.00	0.13	2.76	4.57	4.89	4.63	3.00	80.02
3	0.00	0.10	2.67	4.40	4.78	4.66	3.20	80.19
4	0.00	0.79	2.10	4.70	4.88	4.96	2.30	80.27
5	0.00	0.23	2.20	4.50	4.68	4.75	2.40	81.24
6	0.00	1.19	12.20	15.77	15.90	17.57	7.53	29.85
7	0.00	12.76	4.41	9.48	8.12	13.70	4.77	46.77
8	1.40	5.35	6.64	6.60	5.90	7.52	2.55	64.07
9	6.22	23.85	16.77	12.62	10.01	8.14	2.94	19.45
10	6.29	24.12	16.96	12.76	10.12	8.23	2.97	18.54
11	0.00	23.63	16.61	12.50	9.91	8.07	2.91	26.37
12	0.00	23.43	16.47	12.40	9.83	8.00	2.89	26.98
13	0.00	14.00	9.84	7.40	5.87	8.43	1.72	52.73

## 4 Conclusion

To the best of our knowledge, this study is the first to investigate the effect of effervescent‐tablet‐based nanofluids in the oil‐recovery process. Effervescent‐tablets were prepared by compressing a homogeneous mixture of MWCNTs, SDS, Na_2_CO_3_, and NaH_2_PO_4_ at various concentrations. The Na_2_CO_3_ and NaH_2_PO_4_ included in the as‐consolidated tablets are effervescent agents that generate CO_2_ bubbles when immersed in water; these bubbles provide the buoyancy force required to disperse the nanomaterial and form a suspension. In addition, conventional MWCNT‐based nanofluids (0.01–0.10 vol%) were fabricated though the commonly used two‐step approach. Both categories of nanofluid (effervescent‐tablet‐based and conventional) were characterized in terms of their thermal conductivities and physical stabilities, which revealed that the nanofluid fabricated using high MWCNT and effervescent‐agent concentrations exhibited the highest thermal conductivity (0.701 W m^−1^ K^−1^) among the as‐prepared suspensions. Moreover, the highest and lowest as‐prepared nanofluid thermal conductivities differed by only 10.35%. Thermal conductivity was observed to increase with increasing dispersed‐particle concentration for the conventional nanofluids, which is a trend commonly reported in the literature. However, the thermal properties of the effervescent‐tablet‐based nanofluids were found to depend strongly on effervescent‐agent concentration, which was evident when comparing as‐produced effervescent‐tablet‐based nanofluids with fixed MWCNT but different effervescent‐agent concentrations. The thermal conductivities of the nanofluids prepared with the highest and lowest concentrations of effervescent agent were measured, with the latter observed to provide a 9.53% lower value, which is ascribable to thermal properties that were subsequently found to be linked to dispersed‐particle stability. This conclusion is supported by stability data, which revealed that a high effervescent‐agent concentration leads to better dispersion stability. Nanofluids with similar MWCNT concentrations were also compared, which showed that the hosted nanomaterials are better dispersed and more stable when the effervescent‐tablet technique rather than the conventional fabrication approach is used. The characterized suspensions were subsequently used in a test rig containing a mixture of unconsolidated sand and lower Fares heavy crude oil and water to determine how they influence oil recovery. Thirteen oil recovery process scenarios were explored, including the use of single conventional fluids, steam, and hot water cycles, steam with effervescent‐tablet‐based nanofluids, and steam with conventional nanofluids. Oil‐recovery experiments revealed that the most crude oil was extracted when well‐dispersed nanofluids with high MWCNT concentrations were used in the recovery‐process cycle. Optimum advanced recovery cycling was also observed to increase the amounts of extracted oil by 140.43% and 165.22% over those obtained using optimal conventional recovery cycling and the single fluid flooding recovery approach, respectively. Moreover, the percentage of recovered oil was found to depend strongly on the MWCNT concentration and nanomaterial‐dispersion efficiency when effervescent‐tablet‐based nanofluids were employed in the recovery process. Generally, increasing the MWCNT concentration and enhancing the mixing efficiency increased the amount of extracted oil, and vice versa. The as‐extracted oil was subsequently characterized, which revealed that nanofluid recovery cycling provided lighter oil (i.e., higher API gravities) than those obtained using conventional recovery approaches. In addition, traces of short‐chain hydrocarbons (i.e., C1–C5) were observed in the recovered crude material only when effervescent‐tablet‐based nanofluids with high effervescent‐agent concentrations were included in the recovery cycle. The C1–C5 content was observed to decrease with increasing MWCNT concentration, which is believed to be linked to the CO_2_‐adsorbabilities of the MWCNTs.

In conclusion, the use of effervescent‐tablet‐based nanofluids in the oil recovery process provides a very promising option for the upstream oil industry and has the potential to streamline the logistics of commercial oil production in field applications. The main advantages of the use of such nanofluids are listed below.

1) Unlike conventional nanofluids, effervescent‐tablet‐based nanofluids can be used in situ without requiring additional modifications to existing infrastructure in the field.
2) No sophisticated equipment or high levels of expertise are required to produce these nanofluids; consequently, they are user‐friendly and can easily be adopted for use in EOR applications.
3) The dispersed carbon‐based material used to prepare the effervescent‐tablet‐based nanofluids helps to reduce the viscosity of the oil, which increases its production as a consequence.
4) The accompanying CO_2_ improves oil mobility, thereby increasing its production.

The adoption of such flooding fluids is also associated with some main drawbacks, as summarized below.

1) Nanomaterial agglomeration and sedimentation can occur during operation. Such issue needs to be taken into account because it can cause a clog in the reservoir pores and reduce the effectiveness of the EOR process.
2) Reservoir rocks strongly absorb SDS, which can lead to lower permeability.
3) Some SDS may degrade in the early stages of the reservoir where the temperature is highest; consequently, causing the dispersed carbon‐based materials to cluster, which reduces the effectiveness of the working fluid.
4) Production lines may corrode owing to the accompanying CO_2_ that increases the acidity of the extracted crude oil.

Clearly, further research is required to optimize effervescent‐tablet content based on how it influences the oil‐recovery process. Moreover, the recovery of other types of crude oil needs to be examined to widen the understanding of the influence of such type of suspensions. Also, given that SDS was used as part of the effervescent‐tablet interpretation, potential post‐recovery water treatment measures need to be explored to ensure minimal environmental impact. The impact of using effervescent‐tablet‐based nanofluids on the integrity and permeability of reservoir rocks over time should be explored at different operation conditions. In addition, this category of nanofluid needs to be investigated in an actual oil field in the form of a pilot study to determine any potential obstacles that may be associated with their use before moving toward the commercialization stage.

## Conflict of Interest

The authors declare no conflicts of interest.

## Author Contributions

N.A. led the investigation and was responsible for conceptualization, funding acquisition, project administration, investigation, methodology, supervision, formal analysis, data curation, visualization, resources, writing – original draft, validation, and writing – review and editing. H.B. contributed to conceptualization, investigation, resources, formal analysis, and writing – review and editing. N.F.A. contributed to the investigation, methodology, resources, visualization, and writing, review, and editing. S.A.E. conducted part of the investigation, visualization, writing, review, and editing, and resources. A.T.H. and H.A. participated in resource acquisition, data curation, and investigation. H.B.A., M.A.A., S.K., A.A., M.B., and M.A. contributed to data curation, experimental setup, and investigation. All authors contributed to revising the manuscript and agree with its published version.

## Data Availability

All data can be provided by the author upon reasonable request.
